# Genomic characterization of an ESBL-producing *Klebsiella pneumoniae* ST37 recovered from a hospitalized patient in Armenia

**DOI:** 10.1128/spectrum.00332-25

**Published:** 2025-07-30

**Authors:** Siyu Lu, Jie Sheng, Mary M. Ter-Stepanyan, Yingxiong Wang, Hermine V. Mkrtchyan

**Affiliations:** 1 Department of Paediatrics, Second Affiliated Hospital of the Army Medical University, Chongqing, China; 2 School of Basic Medical Sciences, Chongqing Medical University 12550, Chongqing, China; 3 The Joint International Research Laboratory of Reproduction and Development, Ministry of Education, Chongqing, China; 4 Department of Epidemiology, Faculty of Public Health, Yerevan State Medical University after M. Heratsi, Yerevan, Armenia; 5 Research Center of Maternal and Child Health Protection, Yerevan, Armenia; 6 School of Biomedical Sciences, University of West London 7364, London, United Kingdom; Universita degli Studi dell'Insubria, Varese, Italy

**Keywords:** *Klebsiella pneumoniae*, whole-genome sequencing, ESBL-producing, ST37

## Abstract

Multidrug-resistant *Klebsiella pneumoniae* continues to challenge healthcare services globally. However, our understanding of its occurrence, genomic characteristics, and epidemiology in low- and middle-income countries (LMICs) remains severely limited. In this study, we report the whole-genome sequencing of an extended-spectrum β-lactamase (ESBL)-producing, sequence type (ST) 37 *K*. *pneumoniae* isolate (ARM02) recovered from a patient in Armenia. Antibiotic susceptibility testing revealed that ARM02 was resistant to four of the 11 antibiotics tested, including ampicillin, amoxicillin-clavulanic acid, cefepime, and ceftazidime. Genome sequencing analysis identified seven antimicrobial resistance (AMR) genes in ARM02, including *bla*_TEM-1D_, *bla*_SHV-11_, *dfrA14*, *sul2*, *strA*, *strB*, and the ESBL-producing gene *bla*_CTX-M-15_. In addition, ARM02 harbored 12 virulence genes, including the common pilus fibrillin subunit encoding gene *yagZ/ecpA* and a complete yersiniabactin siderophore system (*irp1*, *irp2*, *ybtAEPQSTUX*, and *fyuA*). Moreover, we also detected five insertion sequences and two plasmid replicons in the ARM02 genome. Phylogenetic analysis showed that ARM02 shared a common ancestor with the USA strains SRR5283489 and SRR5973349, diverging around 2007 (95% onfidence interval, 2004 to 2011). However, ARM02 carried a unique accessory genome, indicating independent evolution and spread. Our findings highlight the co-existence of virulence and resistance genes in the Armenian ST37 strain and emphasize the critical need for genomic surveillance in LMICs. This is crucial for understanding how gram-negative bacterial pathogens, such as ESBL-producing *K. pneumoniae*, which remain on the WHO’s Priority Pathogens list, evolve and spread in LMICs, and how they contribute to the global AMR crisis.

**IMPORTANCE:**

We report the first genomic analysis of an extended-spectrum β-lactamase (ESBL)-producing *Klebsiella pneumoniae* ST37 isolate (ARM02) recovered from a hospitalized patient in Armenia. Genome sequencing identified several antimicrobial resistance (AMR) genes, including *bla*_CTX-M-15_, *bla*_TEM-1D_, and *bla*_SHV-11_, which were directly linked to the observed resistance phenotypes. ARM02 also carried a variety of virulence genes, including a complete yersiniabactin siderophore system, which is associated with enhanced pathogenicity. Phylogenetic analysis revealed that ARM02 was closely related to strains from the United States, but it harbored a unique accessory genome, suggesting independent evolution and local spread in Armenia. This highlights the critical need for robust genomic surveillance and targeted interventions in low- and middle-income countries. This study provides crucial insights into the genetic diversity and potential local transmission of ST37 *K. pneumoniae* in Armenia and calls for larger-scale genomic surveillance to better understand and control its spread.

## INTRODUCTION

Antimicrobial resistance (AMR) continues to challenge healthcare and public services worldwide. In 2019, data from 204 countries showed that 1.27 million deaths were due solely to AMR, surpassing deaths from HIV/AIDS or malaria and that AMR-related infections played a role in 4.95 million deaths (1). *Klebsiella pneumoniae* was among six pathogens, each responsible for more than 250,000 AMR-related deaths, ranking just behind *Staphylococcus aureus* and *Escherichia coli*, marking it an emerging threat to global public health (1). *K. pneumoniae*, a Gram-negative, encapsulated, opportunistic pathogen belonging to the *Enterobacteriaceae* family (2), is ubiquitous and can be found in a wide range of environments, including mucosal surfaces of mammals, soil, and surface water (3). It commonly colonizes hospitalized patients and can cause a variety of infections, such as urinary tract infections, bacteremia, pneumonia, and liver abscesses in immunocompromised individuals (4, 5). Multidrug-resistant strains of *K. pneumoniae*, particularly those extended spectrum β-lactamase (ESBL)-producing strains, are regularly associated with nosocomial outbreaks and pose a serious risk to human health (6). ESBL production is encoded by genes predominantly located on plasmids, with the most common ESBL types being *bla*_TEM_, *bla*_SHV_, and *bla*_CTX-M_, conferring the bacteria to acquire the ability to hydrolyze third-generation cephalosporins (7). Among them, *bla*_CTX-M-15_ and *bla*_CTX-M-14_ are the most widespread variants (8). Mobile genetic elements such as integrons, plasmids, insertion sequences, and transposons contribute greatly to horizontal gene transfer and hence, accelerate the spread of virulence and AMR genes (9).

The global spread of AMR genes has been declared as one of the top ten global public health threats by the World Health Organization (WHO) (10). Low-income and middle-income countries (LMICs) are severely impacted by AMR due to unregulated antimicrobial use, the sale of counterfeit products, and the uncontrolled use of antimicrobials in farming (11, 12). The spread of AMR is a global public health issue, and understanding the molecular epidemiology and drug resistance mechanisms of *K. pneumoniae* will greatly impact clinical treatment options, infection control measures, and public health policies (13). Whole-genome sequencing (WGS) has been instrumental for monitoring and predicting outbreaks caused by pathogens (14). In many high-income countries, WGS has been widely used in studying genomic characteristics, genetic diversity, virulence, and AMR of emerging pathogens, including *K. pneumoniae* (15). However, regions such as Armenia have limited resources to fully utilize such technologies, resulting in a lack of data on the country’s AMR situation and the prevalence of circulating pathogens. Although Armenia developed the AMR National Action Plan in 2015 (16), its practical implementation remains limited. The situation is worsened because there are only a few studies reporting on the WHO’s Priority Pathogens, including ESBL-producing *K. pneumoniae* that maintain their critical status as such pathogens. To our knowledge, previously, only our group reported on WGS analysis of *K. pneumoniae* belonging to ST967 and ST307 recovered from patients in Armenia (17, 18).

In this study, we report for the first time the WGS analysis of *K. pneumoniae* ST37, recovered from the stool sample of a hospitalized patient in Armenia. Further comparative bioinformatics analysis provided insights into the genomic characteristics of this pathogen and shed light on its phylogenetic relatedness with clones found internationally.

## RESULTS

### Isolates and antibiotic susceptibility testing

Eight *K. pneumoniae* isolates belonging to five sequence types (ST37, ST147, ST307, ST807, and ST967) were received from the Medical Microbiology laboratories of three hospitals in Armenia between January 2019 and August 2019. We previously reported the antimicrobial susceptibility profiles of these isolates, along with a comparative genomics analysis of a *K. pneumoniae* ST967 isolate (17). In this study, we report the phylogenomic analysis of the *K. pneumoniae* ST37 isolate, designated ARM02. Antimicrobial susceptibility testing revealed that ARM02 was resistant to the aminopenicillin antibiotic ampicillin, β-lactam antibiotic amoxicillin-clavulanic acid, and the cephalosporin antibiotics cefepime and ceftazidime. However, it remained sensitive to the β-lactam antibiotic piperacillin-tazobactam, fluoroquinolone antibiotics norfloxacin and levofloxacin, the aminoglycoside antibiotic amikacin, and carbapenem antibiotics meropenem and imipenem, as well as chloramphenicol (Table S1).

### Phylogenomic analysis of *K. pneumoniae* ARM02

**Fig 1:**
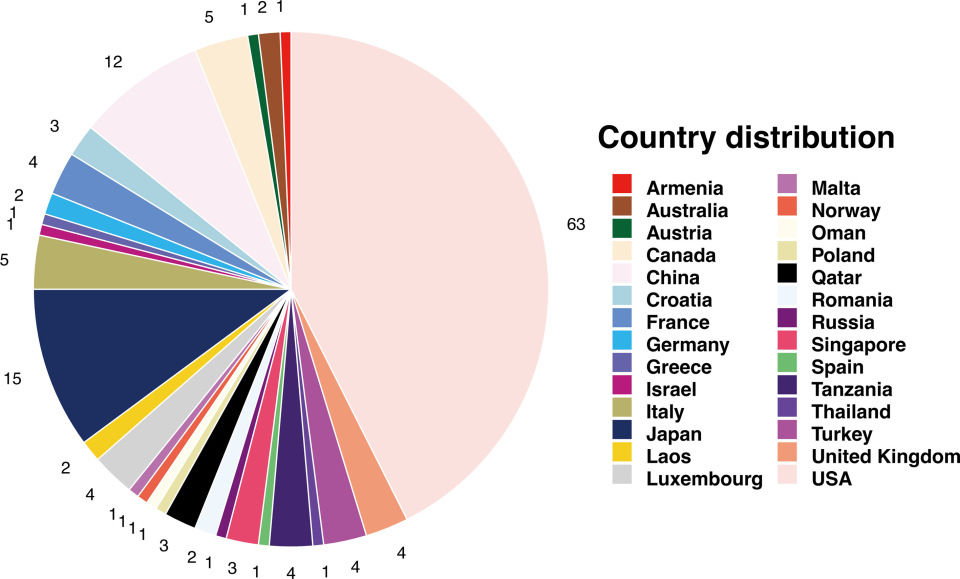
Country origin of *K. pneumoniae* ST37 isolates used in this study.

**Fig 2:**
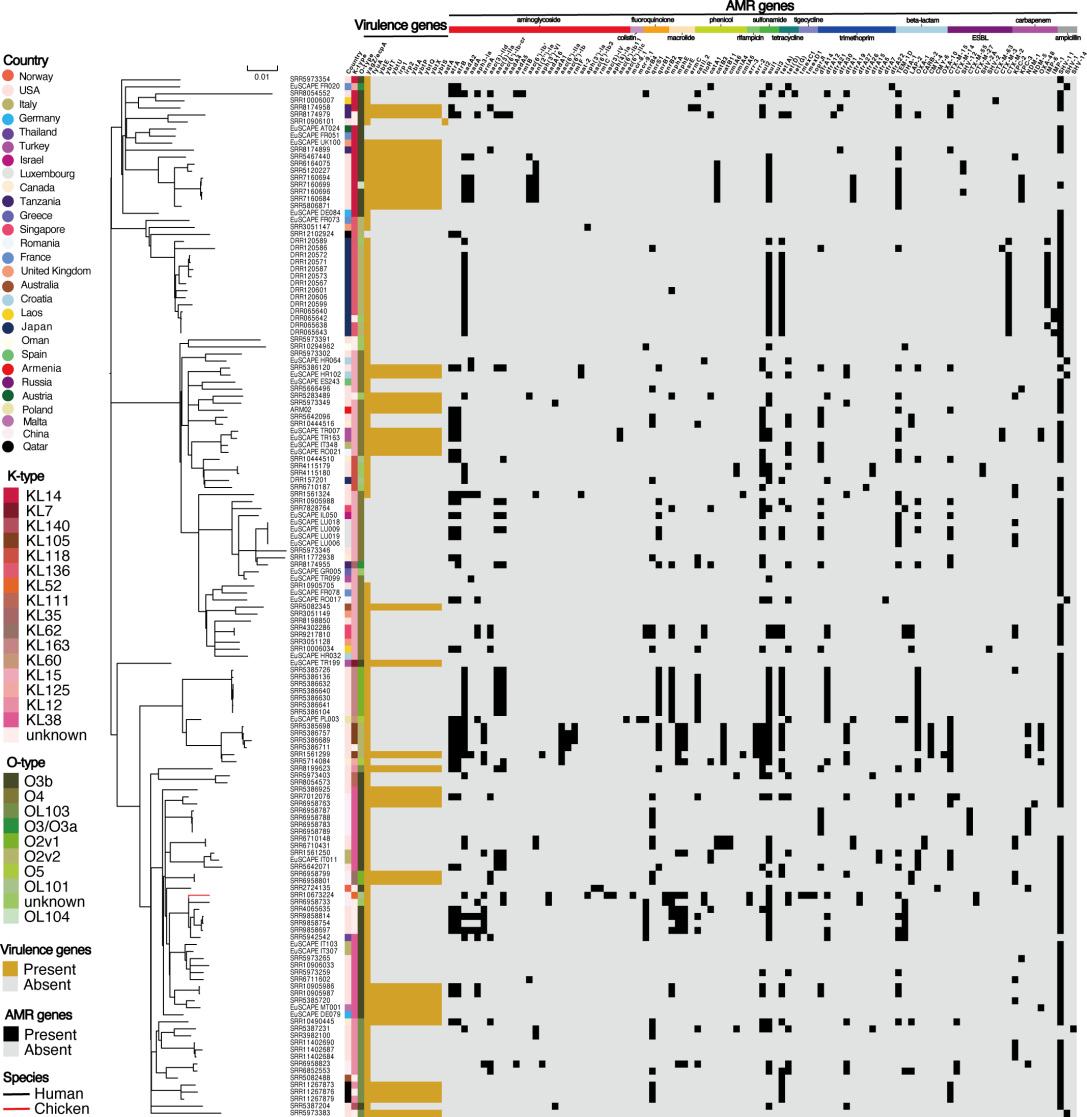
Core SNP phylogenetic analysis of global *K. pneumoniae* ST37 isolates. line color represents the host species. The heatmap columns are as follows: (i) country; (ii) capsule (K) serotype; (iii) lipopolysaccharide (LPS) O-antigen serotype; (iv) virulence genes (orange: present; grey: absent); and (v) AMR genes (black: present; grey: absent).

Multilocus sequence typing (MLST) and serotype analysis revealed that *K. pneumoniae* ARM02 belongs to sequence type 37, capsule type K15, and the O antigen type O4 (Table 1). To delve deeper into the genetic makeup of ARM02, we then compared its genome with 147 publicly available *K. pneumoniae* ST37 genomes. These ST37 isolates were recovered from 28 countries and two sources (human and chicken) (Fig. 1 ; Table S2). The core single-nucleotide polymorphism (SNP) maximum likelihood phylogenetic tree showed that ARM02 was phylogenetically closely related to two human isolates from the United States (SRR5283489 and SRR5973349) (Fig. 2). Pairwise SNP distances analysis of the core genomes revealed that ARM02 had the shortest SNP distance of 216 to SRR5973349, while 243 SNP differences were observed between ARM02 and SRR5283489 (Table S3). The pangenome analysis identified 4,063 core genes (>99% of the pangenome) and 15,032 accessory genes (<99% of the pangenome). Through assessing the accessory genes, we found that ARM02 had a unique accessory genome, despite its close relatedness with SRR5973349 (r = 0.61) (Fig. S1; Table S4). Additionally, the genome-wide average nucleotide identity analysis showed that the Hadamard value between ARM02 and SRR5973349 was 0.955, indicating a high level of genetic similarity (Fig. S2).

**TABLE 1:** Genomic characteristics of ARM02^*a*^^,^^*b*^

Antibiotics, virulence factors, or molecular data		Gene type or molecular data
Antimicrobial resistance		
Beta-lactams		TEM-1D
ESBLs^*a*^		CTX-M-15
Ampicillin		SHV-11
Trimethoprim		dfrA14
Sulfonamides		sul2
Aminoglycosides		strA , strB
Virulence factors profile		
Hypermucoidy (rmpA and/or rmpA2)^*b*^		−
Yersiniabactin (ybt)		ybtS, ybtX, ybtQ, ybtP, ybtA, irp2, irp1, ybtU, ybtT, ybtE, fyuA
Colibactin (clb)		−
Salmochelin (iro)		−
Aerobactin (iuc)		−
Others		yagZ/ecpA
Molecular data		
MLST		ST37
Capsule (K) serotype		KL15
O antigen (lipopolysaccharide) serotype		O4
Plasmid replicon		IncN, IncFIB(K)
Insertion sequence		IS*Ec*9, IS*Kox*1, IS*Kpn*1, IS*6100*, IS*26*

^*a*^ ESBLs, extended spectrum β lactamases; −, negative.

^*b*^ Hypermucoidy (rmpA and/or rmpA2) represents the regulator of mucoid phenotype A.

### Genetic origin of *K. pneumoniae* ST37 population

**Fig 3:**
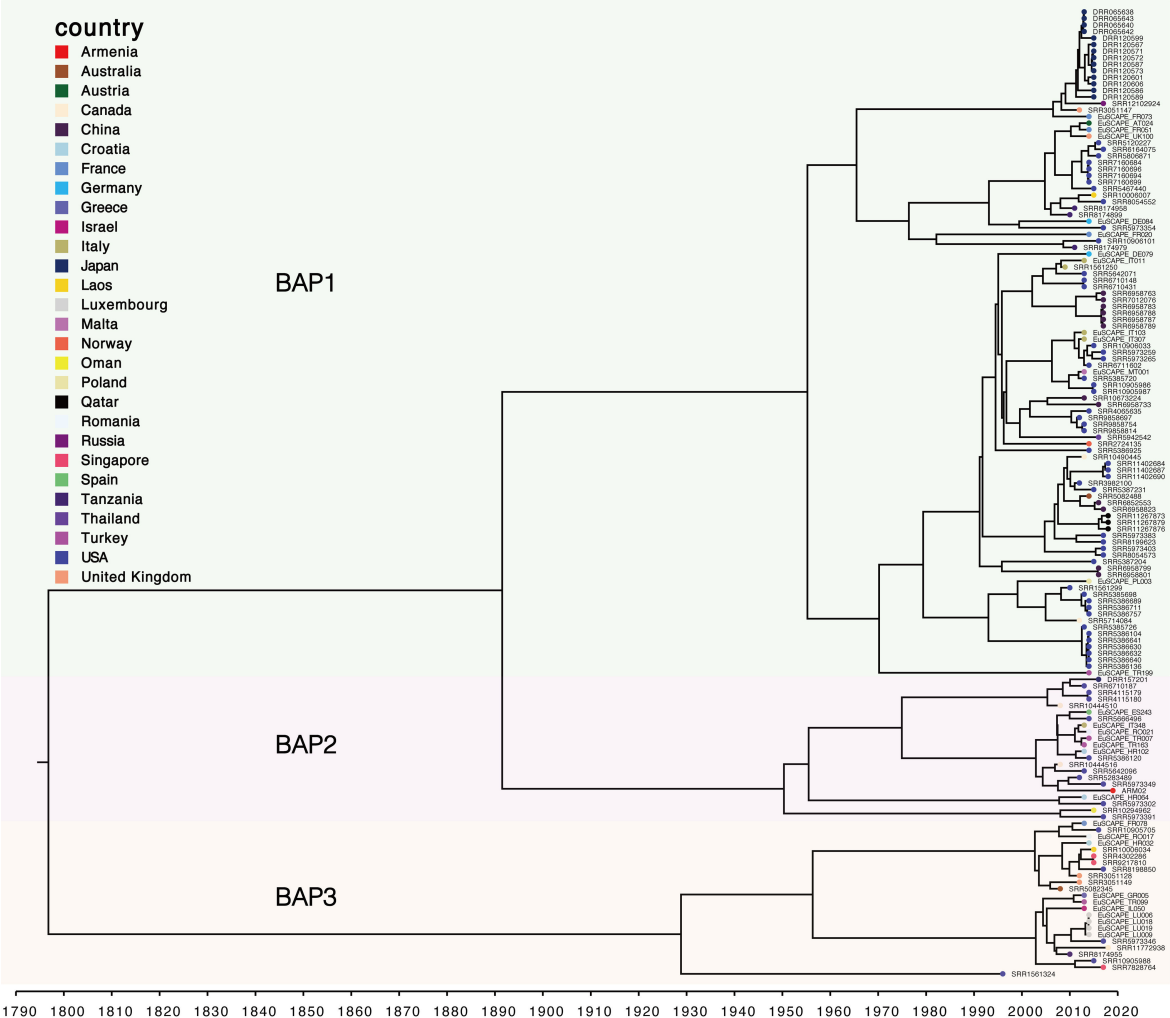
MCC time-calibrated phylogenetic tree of *K. pneumoniae* ST37 isolates. The color of the circle at the end of each branch represents a country. The line color represents the host species. The evolutionary tree was grouped into hree BAP clusters and colored accordingly.

To further investigate the evolutionary origin of the ARM02 and its closely related ST37 isolates, we reconstructed a maximum clade credibility (MCC) tree using BEAST. Results showed that the estimated nucleotide substitution rate for the global ST37 population was 1.43 ×  10^−3^ substitutions per site per year (95% confidence interval [CI], 3.42  ×  10^−4^ to 3.59 ×  10^−3^), with an inferred tree root date of 1796 (95% CI, 1,377 to 1,995) (Fig. 3). Further detailed analyses of the clusters using RhierBAPS revealed that the ST37 isolates could be grouped into three distinct clusters, with ARM02 classified in BAP2. The divergence of ARM02 and its closest related US strains (SRR5973349 and SRR5283489) was inferred to have occurred in 2007 (95% CI, 2,004 to 2,011), indicating that they shared and had descended from a common ancestor.

### Genotypes of resistance and virulence

AMR profiling identified a total of 97 AMR genes across all ST37 isolates, with a median of six AMR genes per isolate (range 0–24) (Table S5). Although ARM02 was phylogenetically related to the strains SRR5973349 and SRR5283489 from the United States, their AMR profiles differed significantly. In the ARM02 genome, we detected 7 AMR genes, including the β-lactam resistance gene *bla*_TEM-1D_, the ESBL-encoding gene *bla*_CTX-M-15_, the ampicillin resistance gene *bla*_SHV-11_, the trimethoprim resistance gene *dfrA14*, the sulfonamides resistance gene *sul2*, and the aminoglycosides resistance genes *strA* and *strB* (Fig. 2 ; Table 1). Among them, *bla*_TEM-1D_, *bla*_CTX-M-15_, *sul2, strA,* and *strB* were located on contig NODE_4, which was identified as plasmid-derived using MOB-suite (Table S6). Several resistance genotypes found in the Armenian isolate ARM02, such as *strA* (38/148), *strB* (38/148), *sul2* (40/148), *bla*_TEM-1D_ (41/148), and *bla*_SHV-11_ (122/148), were also commonly identified in other *K. pneumoniae* ST37 isolates. In addition to the genes detected in ARM02, *aadA2* (45/148), *sul1* (69/148), and *tet(A)* (45/148) were also frequently observed in ST37 isolates. Overall, the ESBL genes were identified in 41% (61/148) of the *K. pneumoniae* ST37 isolates, including the Armenian strain ARM02. Ten different ESBL genes were identified, with *bla*_CTX-M-15_ (25/148) being the most prevalent, followed by *bla*_CTX-M-2_ (13/148) and *bla*_CTX-M-14_ (5/148). However, neither SRR5973349 nor SRR5283489 carried any ESBL genes. Moreover, 36.4% (45/148) of the ST37 strains, including SRR5283489, carried carbapenem-resistant genes; however, no carbapenem resistance genes were detected in ARM02. This was consistent with conventional antibiotic susceptibility testing, which showed that ARM02 was sensitive to the carbapenem antibiotics imipenem and meropenem.

In total, we identified 12 virulence genes in the *K. pneumoniae* ST37 strains included in this study. Among them, *yagZ/ecpA* was present in 133 out of 149 isolates, including ARM02 (Fig. 2; Table S7). Additionally, 40 ST37 isolates, including ARM02, SRR5973349, and SRR5283489, harbored the siderophore yersiniabactin (ybt) locus, which consists of 11 genes (*irp1*, *irp2*, *ybtAEPQSTUX*, and *fyuA*). Notably, only one strain, SRR10906101, was found to carry the *astA* gene encoding heat-stable enterotoxin 1 (East1), which has been commonly reported in *E. coli* isolates. Fifteen isolates did not carry any of the virulence-associated genes tested.

### Mobile genetic elements

**Fig 4:**
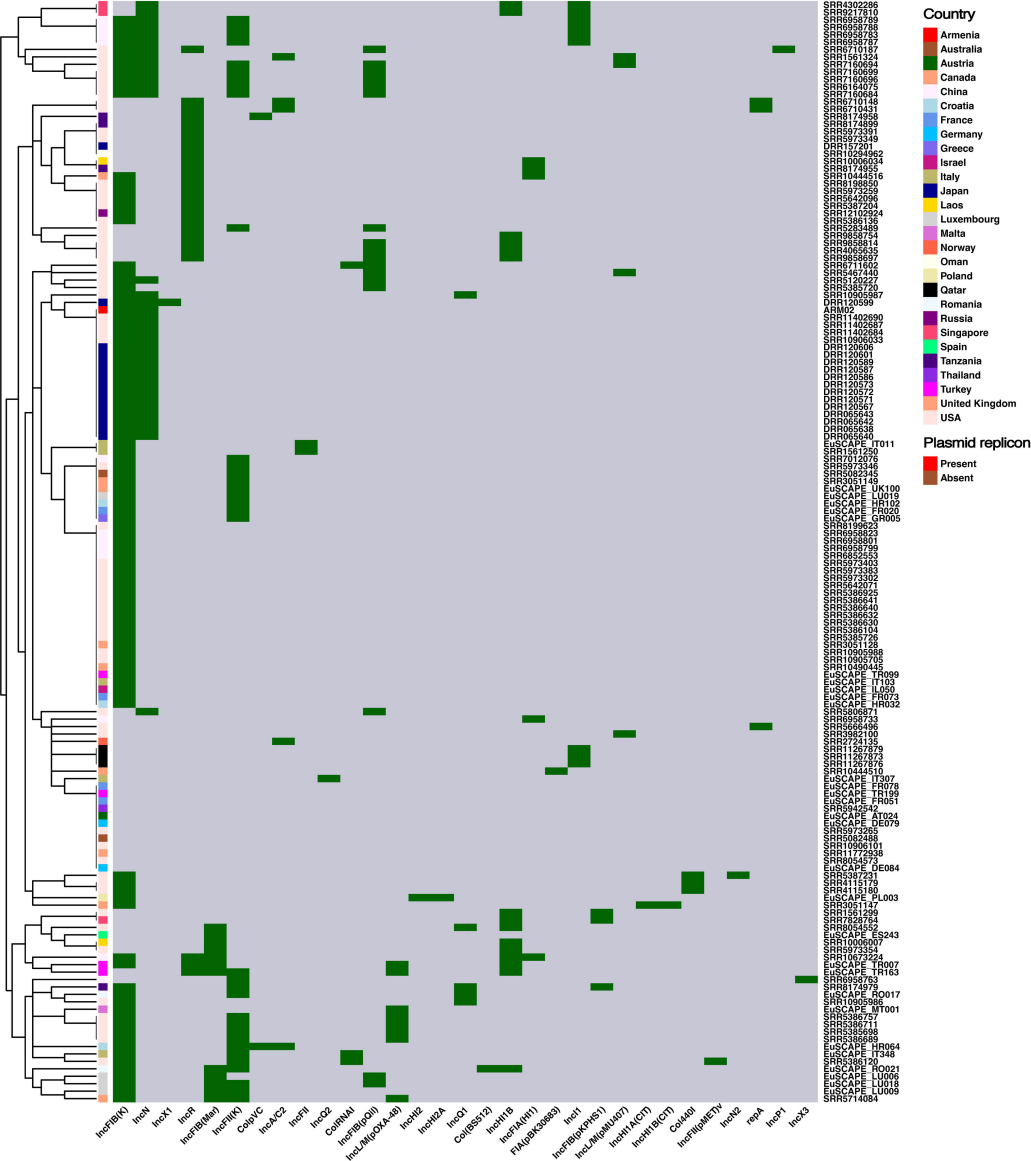
The hierarchical clustering heatmap of plasmid replicon profiles (green: present; grey: absent). The color of the annotation bar indicates the country of origin of the sample.

A total of 31 plasmid replicons were identified in 91.9% (136/148) of *K. pneumoniae* ST37 isolates, with an average number of two replicon types per isolate (Fig. 4; Table S8). ARM02 had a different plasmid replicon profile compared to SRR5973349 and SRR5283489. ARM02 harbored two plasmid replicons, IncN and IncFIB(K) (Fig. 4 ; Table 1). SRR5283489 carried two plasmid replicons, IncFII(K) and IncFIB(pQil), while SRR5973349 only carries IncR (Fig. 4; Table S8). The most common replicons identified in this study were IncFIB(K) (*n* = 101), followed by IncN (*n* = 35), and IncFII(K) (*n* = 34). Among the IncN group, the most frequently identified plasmid type was IncN[pMLST-5] (*n* = 12), followed by IncN[pMLST-9] (*n* = 7), IncN[pMLST-unknown] (*n* = 6), IncN[pMLST-7] (*n* = 5), and IncN[pMLST-15] (*n* = 4). ARM02 carried a unique IncN plasmid, IncN[pMLST-6] (Fig. S3). In addition, we detected five insertion sequences in the ARM02 genome, including IS*Ec*9, IS*Kox*1, IS*Kpn*1, IS*6100*, and IS*26* (Table S9). IS*Ec*9 was found adjacent to the ESBL-gene *bla*_CTX-M-15_.

## DISCUSSION

The first recorded *K. pneumoniae* ST37 strain was identified in the United States in 1996 (19). Since then, ST37 strains have been reported in both human patients and animals worldwide, with the majority of cases reported in Europe (20), the United States (21), and China (22). However, there is currently a dearth of knowledge regarding antibiotic resistance and the genomic characterization of *K. pneumoniae* ST37 in Armenia. In this study, we report for the first time the resistome profile, virulence determinants, and mobile genetic elements of a *K. pneumoniae* ST37 strain, designated ARM02, recovered from a hospitalized patient in Armenia and present its relatedness to *K. pneumoniae* ST37 strains globally.

Antibiotic susceptibility testing revealed that ARM02 was resistant to four of the 11 antibiotics tested. Our results showed a strong correlation between AMR genes and resistance phenotypes: the ampicillin resistance was associated with chromosomally located *bla*_SHV-11_, resistance to amoxicillin-clavulanic acid was linked to *bla*_TEM-1D_, and resistance to cephalosporins (ceftazidime and cefepime) was connected to the ESBL gene *bla*_CTX-M-15_. This highlights the consistency between the detected AMR genes and antimicrobial susceptibility testing results. ESBL-producing *K. pneumoniae* strains are becoming increasingly prevalent worldwide and are classified as “critical priority”-resistant bacteria due to the high morbidity and mortality rates they cause (23). Among the ESBL genes, CTX-M family has emerged as the most widespread, particularly across various genera of Enterobacterales, with *bla*_CTX-M-15_ being the most frequently reported (24, 25). Between 2012 and 2015, it was reported that CTX-M-type ESBLs accounted for 88.7% (1,797/2,025) of all ESBLs identified from 26 medical centres across six Latin American countries (26). In this study, 61 of 148 isolates carried ESBL genes. CTX-M-type ESBLs accounted for 91.8% (56/61) of all ESBLs, with *bla*_CTX-M-15_ being the most common, representing 45.9% (28/61) of the ESBLs identified. Consistent with other studies (27), we found that the *bla*_CTX-M-15_ gene co-occurred with two non-ESBL variants (*bla*_TEM-1D_ and *bla*_SHV-11_) in the ARM02 genome, which is alarming. Using MOB-suite, we identified that *bla*_TEM-1D_, *bla*_CTX-M-15_, *sul2*, *strA*, and *strB* were located on contig NODE_4, which was predicted to be plasmid-derived. Additionally, an insertion sequence element, IS*Ec9*, was found to be located adjacent to *bla*_CTX-M-15_, which may facilitate the horizontal transfer of this AMR gene. Previous studies have suggested that both IS*Ec9* and IncF plasmids play key roles in the global mobilization and dissemination of *bla*_CTX-M-15_ (28–30). Moreover, we identified two plasmid replicons, IncN and IncFIB(K), in the ARM02 genome. IncN plasmids have been previously reported to contribute to the spread of AMR genes among *Enterobacteriaceae* bacteria (31). Furthermore, we detected the presence of an IS26 insertion in the ARM02 genome. Previous studies have shown that the presence of IS*26* in the upstream noncoding regions of *bla*_SHV-1_, *bla*_SHV-2a_, and *bl_a_*_SHV-12_ may promote the acquisition and evolution of an ESBL-positive phenotype (32, 33).

ARM02 was found to harbor 12 virulence genes, including the common pilus fibrillin subunit-encoding gene *yagZ/ecpA*, as well as a complete yersiniabactin siderophore system (*irp1*, *irp2*, *ybtAEPQSTUX*, and *fyuA*). Iron is an essential nutrient needed for bacterial metabolic processes and is acquired via a siderophore-mediated system (34). Ybt is a phenolate siderophore that was first identified in *Yersinia enterocolitica* (35) and has since been commonly found in *Enterobacteriaceae* bacteria (36, 37). Ybt is present in roughly a third of clinical *K. pneumoniae* isolates and is reported to have a significant association with strains isolated from severe infections such as liver abscesses (38, 39). A previous study using a signature-tagged mutagenesis screen identified that an insertional disruption in ybtQ resulted in an attenuated *K. pneumoniae* strain (40, 41). The deletion of YbtPQ in *K. pneumoniae* increases antimicrobial susceptibility. Conversely, the introduction of ICE*Kp* or a plasmid encoding YbtPQ into *E. coli* decreases its susceptibility to a broad range of antimicrobials, suggesting that YbtPQ, located on the mobile genetic element ICE*Kp*, may also facilitate antimicrobial efflux (42). Gu et al. (43) demonstrated that the convergence of virulence and AMR in *K. pneumoniae* isolates represents a significant threat to the treatment and management of *K. pneumoniae* infections, posing a substantial risk to human health due to their hypervirulence, multidrug resistance, and highly transmissible nature (43). Our study highlights the worrying co-existence of virulence and resistance genes in the Armenian *K. pneumoniae* ST37 isolate, emphasizing the importance of genomic surveillance in LMICs.

Phylogenetic analysis showed that ARM02 was most closely related to two USA isolates (SRR5283489 and SRR5973349), with the most recent divergence date of 2007 (95% CI, 2,004 to 2,011), indicating that they shared a common ancestor. ARM02 exhibited the shortest SNP distance of 216 to the US strain SRR5973349 and carried the same virulence factors, indicating that they showed high similarity in the core genome. pMLST schemes allow us to differentiate between plasmids within incompatibility groups and to define epidemiological and evolutionary relatedness. The pMLST result showed that ARM02 carried a unique IncN plasmid, IncN[pMLST-6], which differs from the IncN plasmids found in other ST37 strains. Through assessing the accessory genes, we found that ARM02 possessed a unique accessory genome, although it was most similar to the US strain SRR5973349 (r = 0.61). Despite sharing a common ancestor with the US strains, ARM02 may have evolved and spread independently. Our analysis suggested that ARM02 could be a local strain, not directly transmitted from other countries.

### Conclusions

The main limitation of this study is the small size of the characterized isolates. However, this study provides the first genomic characterization of *K. pneumoniae* ST37 in Armenia, revealing that the strain is resistant to multiple antibiotics and carries several resistance genes, such as *bla*_CTX-M-15_, *bla*_TEM-1D_, and *bla*_SHV-11_, which correlate strongly with its resistance phenotype. The study also identifies virulence-related genes, including a complete yersiniabactin siderophore system, which may contribute to the severity of infections. Phylogenetic analysis showed that ARM02 is closely related to strains from the United States; however, ARM02 possessed a unique accessory genome, suggesting potential independent evolution and spread in Armenia. This highlights the need for local surveillance and implementation of appropriate infection prevention and control meseaures. Although this study reports the genomic analysis of only a single isolate, it provides important insights into the ST37 in Armenia, confirming that larger genomic surveillance studies are needed to further understand its evolution and spread.

## MATERIALS AND METHODS

### Identification and antibiotic susceptibility testing

*K. pneumoniae* ST37 described in this study was among the eight *K. pneumoniae* isolates received from the Medical Microbiology laboratories of three hospitals in Armenia between January 2019 and August 2019 and reported previously (17, 18). All isolates were speciated as *K. pneumoniae* using a matrix-assisted laser desorption ionization-time of flight mass spectrometry as described previously (44) and tested for their susceptibility to eleven antibiotics commonly used in clinical settings in Armenia as described previously (17).

### WGS and molecular analysis

Genomic DNA was extracted using the TIANamp Bacteria DNA Kit (Tiangen, China) according to the manufacturer’s instruction. The DNA that passed quality control was then subjected to library construction using the Nextera XT DNA Sample Preparation kits or TruSeq DNA HT Sample Prep Kit (Illumina, USA). Sequencing was performed on the Illumina HiSeq platform with 151 bp paired-end reads. Quality-control analyses were performed using FASTQC v0.11.9, and adapter sequences were removed using Trimmomatic v0.38 (45). Contig sequences were assembled using SPAdes (version 3.9.0) (46). The annotation was performed with Prokka v1.14.5 (47). Sequence types of *K. pneumoniae* isolates were determined by MLST v2.19.0 (https://github.com/tseemann/mlst) (48). Capsule serotype, O antigen, and AMR genes (> 90% identity and 90% coverage) were detected using Kleborate v0.3.0 (https://github.com/katholt/Kleborate) (49). The MOB-suite (https://github.com/phac-nml/mob-suite) was used to determine which antibiotic resistance genes are encoded on plasmids (50). Plasmid replicon types were identified using Abricate v1.0.1 (https://github.com/tseemann/abricate) through the PlasmidFinder database (51) with a minimum identity of 90% and minimum coverage length of 60%. Plasmid typing was done in pMLST v1.4 (https://cge.food.dtu.dk/services/pMLST/). Abricate v1.0.1 (https://github.com/tseemann/abricate) was used to predict virulence genes (> 90% identity and 90% coverage) profiling through the VFDB database (https://github.com/haruosuz/vfdb).

### Core and accessory genome analysis

Previously reported *K. pneumoniae* ST37 genomes were downloaded from the Pathogenwatch database (52) (https://pathogen.watch). All isolates were aligned to the reference genome (GenBank accession number: CP060049.1), and SNPs were identified using Snippy v.4.6.0 (https://github.com/tseemann/snippy). Gubbins v2.4.1 (53) was used to detect recombinant regions. We constructed a maximum likelihood core SNP phylogenetic tree using FastTree v2.1.11 (54), with 100 bootstraps and the GTR + GAMMA model. The trees were visualized and optimized via Evolview (55, 56). Hierarchically clustering of the sequence data to reveal nested population structure was applied using RhierBAPS (57). Pairwise SNP differences were assessed using the snp-dists tool (https://github.com/tseemann/snp-dists). Average nucleotide identities were computed using the software Pyani v0.2.11 (https://github.com/widdowquinn/pyani) (58). The core and accessory genomes were calculated using the rapid standalone pangenomic pipeline Roary v3.13.0 (59) (https://sanger-pathogens.github.io/Roary).

### Time-calibrated phylogenetic reconstruction

Bayesian time-scaled phylogenetic analysis was performed using Bayesian evolutionary analysis sampling trees (BEAST v2.6.3) (60). The best-fitting nucleotide substitution model was determined using jModelTest v2.1.10 (61). A Bayesian skyline coalescent model and “Relaxed Clock Log Normal” molecular clock were selected. GTR with gamma substitution model (GTR + Gamma) and a rapid bootstrap procedure (100 replicates) were employed. The Markov chain Monte Carlo (MCMC) chain was run for 10 million generations and sampled every 1,000th generation. The convergence of MCMC analyses was diagnosed in the Tracer v1.7.2. MCC tree was generated using TreeAnnotator (https://beast.community/treeannotator) and visualized by FigTree v1.4.4 (http://tree.bio.ed.ac.uk/software/figtree/).

## Data Availability

WGS data for this work were deposited in the ENA database under the accession number ERR9882336. Public *K. pneumoniae* ST37 genomes were downloaded from the Pathogenwatch database (details were provided in Table S2).

## SUPPLEMENTAL MATERIAL

Figure S1 spectrum.00332-25-s0001.tif Hierarchical clustering heatmap of the accessory genome of *Klebsiella pneumoniae* ST37 isolates.

Figure S2 spectrum.00332-25-s0002.tif Hierarchical clustering based on pairwise ANI comparisons of all *Klebsiella pneumoniae* ST37 isolates.

Figure S3 spectrum.00332-25-s0003.tif Heatmap of IncN pMLST profiles.

Supplemental tables spectrum.00332-25-s0004.xlsx Tables S1 to S8.

ASM does not own the copyrights to Supplemental Material that may be linked to, or accessed through, an article. The authors have granted ASM a non-exclusive, world-wide license to publish the Supplemental Material files. Please contact the corresponding author directly for reuse.

## References

[1] Murray CJL, Ikuta KS, Sharara F, Swetschinski L, Robles Aguilar G, Gray A, Han C, Bisignano C, Rao P, Wool E, et al.. 2022. Global burden of bacterial antimicrobial resistance in 2019: a systematic analysis. The Lancet 399:629–655. doi:10.1016/S0140-6736(21)02724-0 PMC8841637 35065702

[2] Friedländer C. 1882. Ueber die Schizomyceten bei der acuten fibrösen Pneumonie. Archiv für pathologische Anatomie und Physiologie und für klinische Medicin 87:319–324. doi:10.1515/9783112404508-017

[3] Martin RM, Bachman MA. 2018. Colonization, infection, and the accessory genome of Klebsiella pneumoniae Front Cell Infect Microbiol 8:4. doi:10.3389/fcimb.2018.00004 29404282 PMC5786545

[4] Fung CP, Lin YT, Lin JC, Chen TL, Yeh KM, Chang FY, Chuang HC, Wu HS, Tseng CP, Siu LK. 2012. Klebsiella pneumoniae in gastrointestinal tract and pyogenic liver abscess. Emerg Infect Dis 18:1322–1325. doi:10.3201/eid1808.111053 22840473 PMC3414011

[5] Paczosa MK, Mecsas J. 2016. Klebsiella pneumoniae: going on the offense with a strong defense. Microbiol Mol Biol Rev 80:629–661. doi:10.1128/MMBR.00078-15 27307579 PMC4981674

[6] Podschun R, Ullmann U. 1998. Klebsiella spp. as nosocomial pathogens: epidemiology, taxonomy, typing methods, and pathogenicity factors. Clin Microbiol Rev 11:589–603. doi:10.1128/CMR.11.4.589 9767057 PMC88898

[7] Paterson DL, Bonomo RA. 2005. Extended-spectrum beta-lactamases: a clinical update. Clin Microbiol Rev 18:657–686. doi:10.1128/CMR.18.4.657-686.2005 16223952 PMC1265908

[8] Bush K, Bradford PA. 2020. Epidemiology of β-lactamase-producing pathogens. Clin Microbiol Rev 33:e00047-19. doi:10.1128/CMR.00047-19 32102899 PMC7048014

[9] Khedkar S, Smyshlyaev G, Letunic I, Maistrenko OM, Coelho LP, Orakov A, Forslund SK, Hildebrand F, Luetge M, Schmidt TSB, Barabas O, Bork P. 2022. Landscape of mobile genetic elements and their antibiotic resistance cargo in prokaryotic genomes. Nucleic Acids Res 50:3155–3168. doi:10.1093/nar/gkac163 35323968 PMC8989519

[10] EClinicalMedicine. 2021. Antimicrobial resistance: a top ten global public health threat. EClinicalMedicine 41:101221. doi:10.1016/j.eclinm.2021.101221 34877512 PMC8633964

[11] Kelesidis T, Falagas ME. 2015. Substandard/counterfeit antimicrobial drugs. Clin Microbiol Rev 28:443–464. doi:10.1128/CMR.00072-14 25788516 PMC4402958

[12] Morgan DJ, Okeke IN, Laxminarayan R, Perencevich EN, Weisenberg S. 2011. Non-prescription antimicrobial use worldwide: a systematic review. Lancet Infect Dis 11:692–701. doi:10.1016/S1473-3099(11)70054-8 21659004 PMC3543997

[13] NIHR Global Health Research Unit on Genomic Surveillance of AMR. 2020. Whole-genome sequencing as part of national and international surveillance programmes for antimicrobial resistance: a roadmap. BMJ Glob Health 5:e002244. doi:10.1136/bmjgh-2019-002244 PMC7689591 33239336

[14] Baker S, Thomson N, Weill FX, Holt KE. 2018. Genomic insights into the emergence and spread of antimicrobial-resistant bacterial pathogens. Science 360:733–738. doi:10.1126/science.aar3777 29773743 PMC6510332

[15] Bentley SD, Parkhill J. 2015. Genomic perspectives on the evolution and spread of bacterial pathogens. Proc Biol Sci 282:20150488. doi:10.1098/rspb.2015.0488 26702036 PMC4707741

[16] Navasardyan N, Harutyunyan T, Abrahamyan L. 2016. Antibiotic use: a cross-sectional survey of knowledge, attitude and practice among Yerevan adult population, Master of Public Health Integrating Experience Project, Yerevan

[17] Sheng J, Cave R, Ter-Stepanyan MM, Kotsinyan N, Chen J, Zhang L, Jiang T, Mkrtchyan HV. 2023. Whole-Genome Sequencing and Comparative Genomics Analysis of a Newly Emerged Multidrug-Resistant Klebsiella pneumoniae Isolate of ST967. Microbiol Spectr 11:e0401122. doi:10.1128/spectrum.04011-22 37022188 PMC10269624

[18] Sheng J, Cave R, Ter-Stepanyan MM, Lu S, Wang Y, Liu T, Mkrtchyan HV. 2024. Emergence of mcr-8.1-bearing MDR-hypervirulent Klebsiella pneumoniae ST307. Microbiol Spectr 13:e01910–24. doi:10.1128/spectrum.01910-24 39670759 PMC11792491

[19] Kitchel B, Rasheed JK, Patel JB, Srinivasan A, Navon-Venezia S, Carmeli Y, Brolund A, Giske CG. 2009. Molecular epidemiology of KPC-producing Klebsiella pneumoniae isolates in the United States: clonal expansion of multilocus sequence type 258. Antimicrob Agents Chemother 53:3365–3370. doi:10.1128/AAC.00126-09 19506063 PMC2715580

[20] Agosta M, Bencardino D, Argentieri M, Pansani L, Sisto A, Ciofi Degli Atti ML, D’Amore C, Bagolan P, Iacobelli BD, Magnani M, Raponi M, Perno CF, Andreoni F, Bernaschi P. 2023. Clonal spread of hospital-acquired NDM-1-producing Klebsiella pneumoniae and Escherichia coli in an Italian neonatal surgery unit: a retrospective study. Antibiotics (Basel) 12:642. doi:10.3390/antibiotics12040642 37107005 PMC10135170

[21] Guo Q, Spychala CN, McElheny CL, Doi Y. 2016. Comparative analysis of an IncR plasmid carrying armA, blaDHA-1 and qnrB4 from Klebsiella pneumoniae ST37 isolates. J Antimicrob Chemother 71:882–886. doi:10.1093/jac/dkv444 26747096 PMC4790621

[22] Yang J, Ye L, Guo L, Zhao Q, Chen R, Luo Y, Chen Y, Tian S, Zhao J, Shen D, Han L. 2013. A nosocomial outbreak of KPC-2-producing Klebsiella pneumoniae in a Chinese hospital: dissemination of ST11 and emergence of ST37, ST392 and ST395. Clin Microbiol Infect 19:E509–E515. doi:10.1111/1469-0691.12275 23841705

[5] Tacconelli E, Carrara E, Savoldi A, Harbarth S, Mendelson M, Monnet DL, Pulcini C, Kahlmeter G, Kluytmans J, Carmeli Y, et al.. 2018. Discovery, research, and development of new antibiotics: the WHO priority list of antibiotic-resistant bacteria and tuberculosis. Lancet Infect Dis 18:318–327. doi:10.1016/S1473-3099(17)30753-3 29276051

[24] Bevan ER, Jones AM, Hawkey PM. 2017. Global epidemiology of CTX-M β-lactamases: temporal and geographical shifts in genotype. J Antimicrob Chemother 72:2145–2155. doi:10.1093/jac/dkx146 28541467

[25] Pitout JD, Laupland KB. 2008. Extended-spectrum β-lactamase-producing Enterobacteriaceae: an emerging public-health concern. Lancet Infect Dis 8:159–166. doi:10.1016/S1473-3099(08)70041-0 18291338

[26] Karlowsky JA, Kazmierczak KM, Bouchillon SK, de Jonge BLM, Stone GG, Sahm DF. 2019. In Vitro Activity of Ceftazidime-Avibactam against Clinical Isolates of Enterobacteriaceae and Pseudomonas aeruginosa collected in Latin American Countries: results from the INFORM Global Surveillance Program, 2012 to 2015. Antimicrob Agents Chemother 63:e01814-18. doi:10.1128/AAC.01814-18 30670424 PMC6437529

[27] Liakopoulos A, Mevius D, Ceccarelli D. 2016. A review of SHV extended-spectrum β-lactamases: neglected yet ubiquitous. Front Microbiol 7:1374. doi:10.3389/fmicb.2016.01374 27656166 PMC5011133

[28] Mbelle NM, Feldman C, Osei Sekyere J, Maningi NE, Modipane L, Essack SY. 2019. The resistome, mobilome, virulome and phylogenomics of multidrug-resistant Escherichia coli clinical isolates from Pretoria, South Africa. Sci Rep 9:16457. doi:10.1038/s41598-019-52859-2 31712587 PMC6848087

[29] Osei Sekyere J, Maningi NE, Modipane L, Mbelle NM. 2020. Emergence of mcr-9.1 in extended-spectrum-β-Lactamase-producing clinical Enterobacteriaceae in Pretoria, South Africa: Global evolutionary phylogenomics, resistome, and mobilome . mSystems 5. doi:10.1128/msystems.00148-20 PMC7253365 32430406

[30] Frenk S, Rakovitsky N, Temkin E, Schechner V, Cohen R, Kloyzner BS, Schwaber MJ, Solter E, Cohen S, Stepansky S, Carmeli Y. 2020. Investigation of outbreaks of extended-spectrum beta-lactamase-producing Klebsiella pneumoniae in three neonatal intensive care units using whole genome sequencing. Antibiotics (Basel) 9:705. doi:10.3390/antibiotics9100705 33081087 PMC7650633

[31] Carattoli A. 2009. Resistance plasmid families in Enterobacteriaceae. Antimicrob Agents Chemother 53:2227–2238. doi:10.1128/AAC.01707-08 19307361 PMC2687249

[32] Hammond DS, Schooneveldt JM, Nimmo GR, Huygens F, Giffard PM. 2005. Bla(SHV) Genes in Klebsiella pneumoniae: different allele distributions are associated with different promoters within individual isolates. Antimicrob Agents Chemother 49:256–263. doi:10.1128/AAC.49.1.256-263.2005 15616303 PMC538876

[33] Kim J, Shin HS, Seol SY, Cho DT. 2002. Relationship between blaSHV-12 and blaSHV-2a in Korea. J Antimicrob Chemother 49:261–267. doi:10.1093/jac/49.2.261 11815566

[34] Lill R, Srinivasan V, Mühlenhoff U. 2014. The role of mitochondria in cytosolic-nuclear iron–sulfur protein biogenesis and in cellular iron regulation. Curr Opin Microbiol 22:111–119. doi:10.1016/j.mib.2014.09.015 25460804

[5] Heesemann J, Hantke K, Vocke T, Saken E, Rakin A, Stojiljkovic I, Berner R. 1993. Virulence of Yersinia enterocolitica is closely associated with siderophore production, expression of an iron‐repressible outer membrane polypeptide of 65 000 Da and pesticin sensitivity . Mol Microbiol 8:397–408. doi:10.1111/j.1365-2958.1993.tb01583.x 8316088

[36] Heffernan J, Wildenthal J, Tran H, Katumba GL, McCoy W, Henderson J. 2024. Yersiniabactin is a quorum-sensing autoinducer and siderophore in uropathogenic Escherichia coli. MBio. doi:10.1128/mbio.00277-23:e0027723 PMC10865836 38236035

[37] Hetland MAK, Hawkey J, Bernhoff E, Bakksjø RJ, Kaspersen H, Rettedal SI, Sundsfjord A, Holt KE, Löhr IH. 2023. Within-patient and global evolutionary dynamics of Klebsiella pneumoniae ST17. Microb Genom 9:mgen001005. doi:10.1099/mgen.0.001005 37200066 PMC10272876

[38] Holt KE, Wertheim H, Zadoks RN, Baker S, Whitehouse CA, Dance D, Jenney A, Connor TR, Hsu LY, Severin J, et al.. 2015. Genomic analysis of diversity, population structure, virulence, and antimicrobial resistance in Klebsiella pneumoniae, an urgent threat to public health. Proc Natl Acad Sci USA 112:E3574–81. doi:10.1073/pnas.1501049112 26100894 PMC4500264

[39] Lam MMC, Wick RR, Wyres KL, Gorrie CL, Judd LM, Jenney AWJ, Brisse S, Holt KE. 2018. Genetic diversity, mobilisation and spread of the yersiniabactin-encoding mobile element ICEKp in Klebsiella pneumoniae populations. Microb Genom 4:e000196. doi:10.1099/mgen.0.000196 29985125 PMC6202445

[40] Lawlor MS, O’connor C, Miller VL. 2007. Yersiniabactin is a virulence factor for Klebsiella pneumoniae during pulmonary infection. Infect Immun 75:1463–1472. doi:10.1128/IAI.00372-06 17220312 PMC1828572

[41] Lawlor MS, Hsu J, Rick PD, Miller VL. 2005. Identification of Klebsiella pneumoniae virulence determinants using an intranasal infection model. Mol Microbiol 58:1054–1073. doi:10.1111/j.1365-2958.2005.04918.x 16262790

[42] Farzand R, Rajakumar K, Barer MR, Freestone PPE, Mukamolova GV, Oggioni MR, O’Hare HM. 2021. A Virulence Associated Siderophore Importer Reduces Antimicrobial Susceptibility of Klebsiella pneumoniae. Front Microbiol 12:607512. doi:10.3389/fmicb.2021.607512 33584611 PMC7876324

[43] Gu D, Dong N, Zheng Z, Lin D, Huang M, Wang L, Chan EW-C, Shu L, Yu J, Zhang R, Chen S. 2018. A fatal outbreak of ST11 carbapenem-resistant hypervirulent Klebsiella pneumoniae in a Chinese hospital: a molecular epidemiological study. Lancet Infect Dis 18:37–46. doi:10.1016/S1473-3099(17)30489-9 28864030

[44] Mkrtchyan HV, Russell CA, Wang N, Cutler RR. 2013. Could public restrooms be an environment for bacterial resistomes? PLoS One 8:e54223. doi:10.1371/journal.pone.0054223 23349833 PMC3547874

[45] Bolger AM, Lohse M, Usadel B. 2014. Trimmomatic: a flexible trimmer for Illumina sequence data. Bioinformatics 30:2114–2120. doi:10.1093/bioinformatics/btu170 24695404 PMC4103590

[46] Prjibelski A, Antipov D, Meleshko D, Lapidus A, Korobeynikov A. 2020. Using SPAdes de novo assembler. CP in Bioinformatics 70. doi:10.1002/cpbi.102 32559359

[47] Seemann T. 2014. Prokka: rapid prokaryotic genome annotation. Bioinformatics 30:2068–2069. doi:10.1093/bioinformatics/btu153 24642063

[48] Jolley KA, Bray JE, Maiden MCJ. 2018. Open-access bacterial population genomics: BIGSdb software, the PubMLST.org website and their applications. Wellcome Open Res 3:124. doi:10.12688/wellcomeopenres.14826.1 30345391 PMC6192448

[49] Lam MMC, Wick RR, Watts SC, Cerdeira LT, Wyres KL, Holt KE. 2021. A genomic surveillance framework and genotyping tool for Klebsiella pneumoniae and its related species complex. Nat Commun 12:4188. doi:10.1038/s41467-021-24448-3 34234121 PMC8263825

[50] Robertson J, Nash JHE. 2018. MOB-suite: software tools for clustering, reconstruction and typing of plasmids from draft assemblies. Microb Genom 4:e000206. doi:10.1099/mgen.0.000206 30052170 PMC6159552

[51] Carattoli A, Zankari E, García-Fernández A, Voldby Larsen M, Lund O, Villa L, Møller Aarestrup F, Hasman H. 2014. In silico detection and typing of plasmids using PlasmidFinder and plasmid multilocus sequence typing. Antimicrob Agents Chemother 58:3895–3903. doi:10.1128/AAC.02412-14 24777092 PMC4068535

[52] Argimón S, David S, Underwood A, Abrudan M, Wheeler NE, Kekre M, Abudahab K, Yeats CA, Goater R, Taylor B, Harste H, Muddyman D, Feil EJ, Brisse S, Holt K, Donado-Godoy P, Ravikumar KL, Okeke IN, Carlos C, Aanensen DM, NIHR Global Health Research Unit on Genomic Surveillance of Antimicrobial Resistance*.* 2021*.* Rapid genomic characterization and global surveillance of Klebsiella using Pathogenwatch. Clin Infect Dis 73:S325–S335. doi:10.1093/cid/ciab784 34850838 PMC8634497

[53] Croucher NJ, Page AJ, Connor TR, Delaney AJ, Keane JA, Bentley SD, Parkhill J, Harris SR. 2015. Rapid phylogenetic analysis of large samples of recombinant bacterial whole genome sequences using Gubbins. Nucleic Acids Res 43:e15. doi:10.1093/nar/gku1196 25414349 PMC4330336

[54] Price MN, Dehal PS, Arkin AP. 2010. FastTree 2--approximately maximum-likelihood trees for large alignments. PLoS One 5:e9490. doi:10.1371/journal.pone.0009490 20224823 PMC2835736

[55] Zhang H, Gao S, Lercher MJ, Hu S, Chen WH. 2012. EvolView, an online tool for visualizing, annotating and managing phylogenetic trees. Nucleic Acids Res 40:W569–72. doi:10.1093/nar/gks576 22695796 PMC3394307

[56] He Z, Zhang H, Gao S, Lercher MJ, Chen WH, Hu S. 2016. Evolview v2: an online visualization and management tool for customized and annotated phylogenetic trees. Nucleic Acids Res 44:W236–41. doi:10.1093/nar/gkw370 27131786 PMC4987921

[57] Tonkin-Hill G, Lees JA, Bentley SD, Frost SDW, Corander J. 2018. RhierBAPS: An R implementation of the population clustering algorithm hierBAPS. Wellcome Open Res 3:93. doi:10.12688/wellcomeopenres.14694.1 30345380 PMC6178908

[58] Pritchard L, Glover RH, Humphris S, Elphinstone JG, Toth IK. 2016. Genomics and taxonomy in diagnostics for food security: soft-rotting enterobacterial plant pathogens. Anal Methods 8:12–24. doi:10.1039/C5AY02550H

[59] Page AJ, Cummins CA, Hunt M, Wong VK, Reuter S, Holden MTG, Fookes M, Falush D, Keane JA, Parkhill J. 2015. Roary: rapid large-scale prokaryote pan genome analysis. Bioinformatics 31:3691–3693. doi:10.1093/bioinformatics/btv421 26198102 PMC4817141

[60] Drummond AJ, Rambaut A. 2007. BEAST: Bayesian evolutionary analysis by sampling trees. BMC Evol Biol 7:214. doi:10.1186/1471-2148-7-214 17996036 PMC2247476

[61] Darriba D, Taboada GL, Doallo R, Posada D. 2012. jModelTest 2: more models, new heuristics and parallel computing. Nat Methods 9:772. doi:10.1038/nmeth.2109 PMC4594756 22847109

