# Supplementary figures and images for: Genomic characterization of an ESBL-producing Klebsiella pneumoniae ST37 recovered from a hospitalized patient in Armenia
Source: Microbiol Spectr. 2025 Jul 30;13(9):e00332-25. doi: 10.1128/spectrum.00332-25 (PMC12403571; doi:10.1128/spectrum.00332-25)

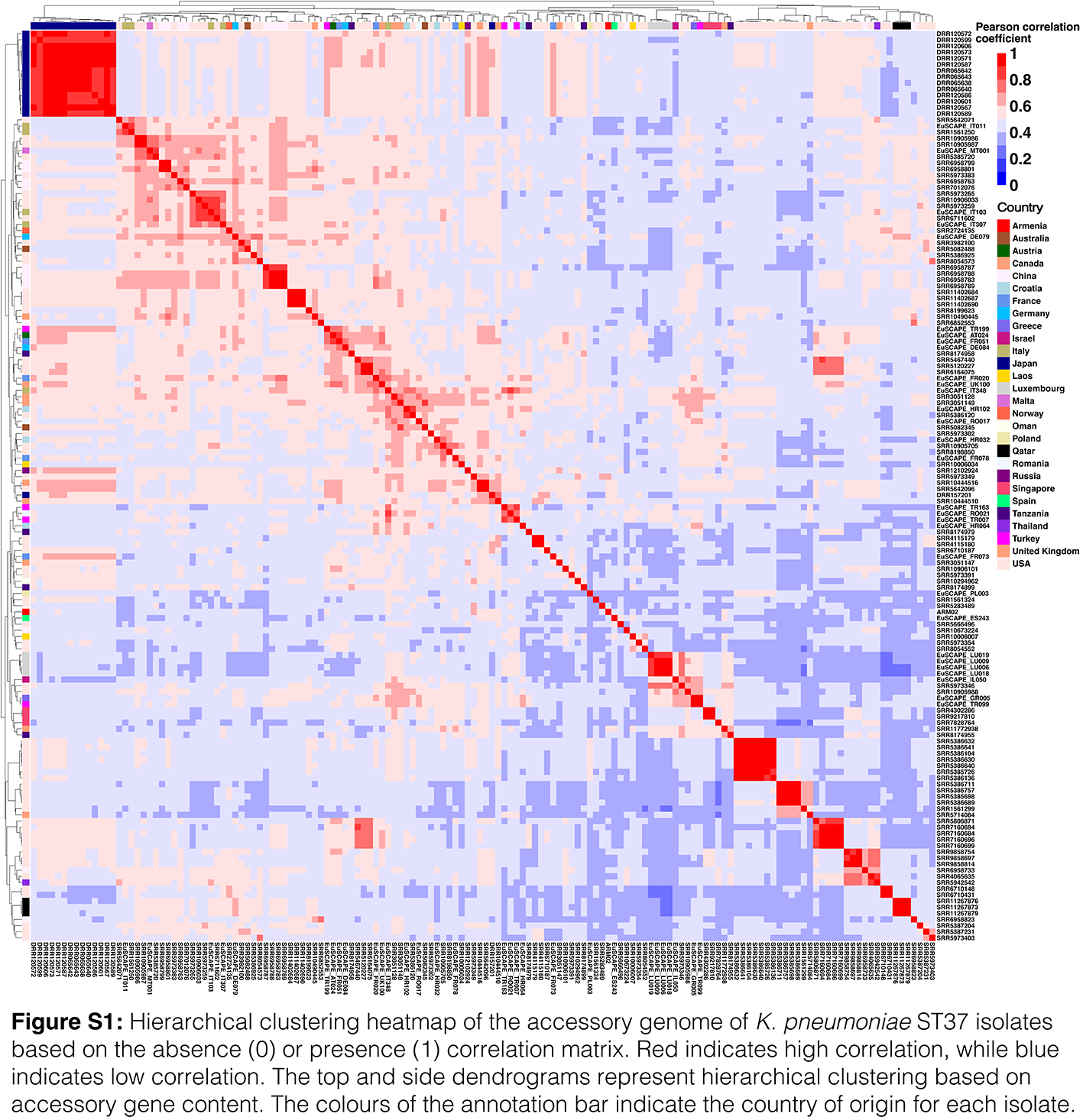

Supplement: Figure S1 — Hierarchical clustering heatmap of the accessory genome of Klebsiella pneumoniae ST37 isolates. [file spectrum.00332-25-s0001.tif]

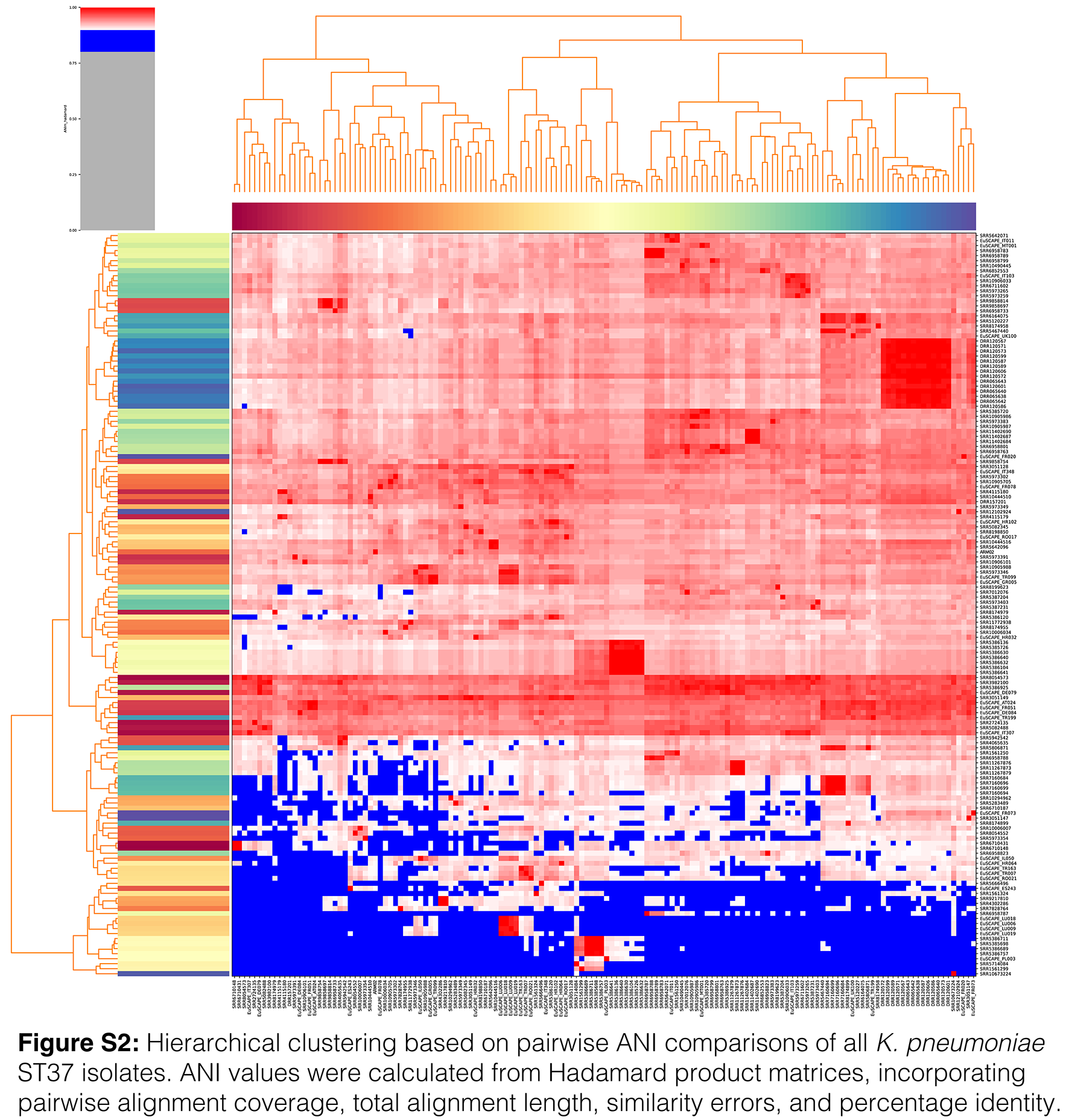

Supplement: Figure S2 — Hierarchical clustering based on pairwise ANI comparisons of all Klebsiella pneumoniae ST37 isolates. [file spectrum.00332-25-s0002.tif]

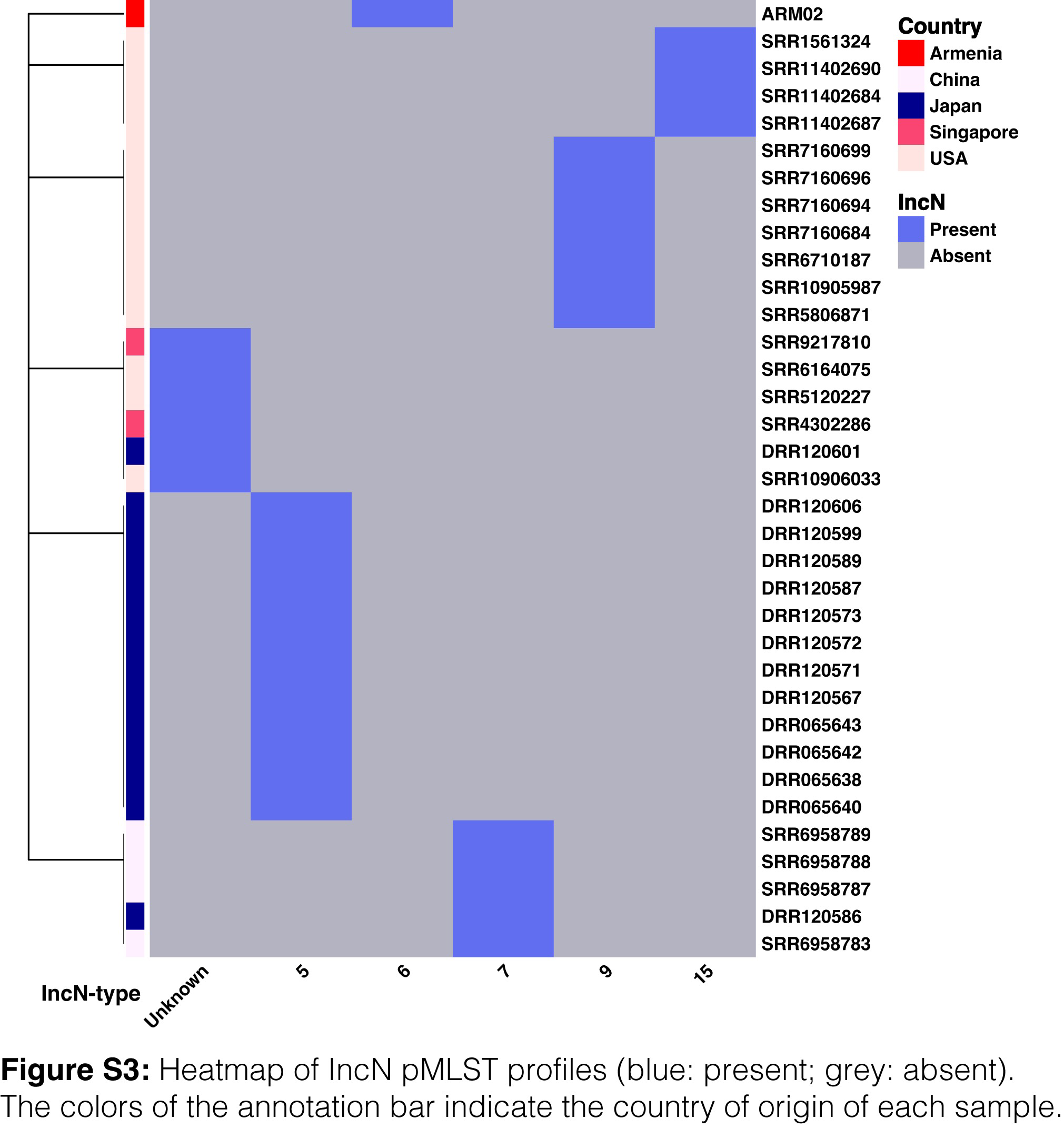

Supplement: Figure S3 — Heatmap of IncN pMLST profiles. [file spectrum.00332-25-s0003.tif]
